# Hyaluronan and Fibrin Biomaterial as Scaffolds for Neuronal Differentiation of Adult Stem Cells Derived from Adipose Tissue and Skin

**DOI:** 10.3390/ijms12106749

**Published:** 2011-10-12

**Authors:** Chiara Gardin, Vincenzo Vindigni, Eriberto Bressan, Letizia Ferroni, Elisa Nalesso, Alessandro Della Puppa, Domenico D’Avella, Diego Lops, Paolo Pinton, Barbara Zavan

**Affiliations:** 1 Department of Histology, Microbiology and Medical Biotechnology, University of Padova, Via G. Colombo 3, 35100 Padova, Italy; E-Mails: Chiaragardin@unipd.it (C.G.); letia.ferroni@unipd.it (L.F.); elisa.nalessoo@unipd.it (E.N.); 2 Unit of Plastic and Reconstructive Surgery, University of Padova, Via Giustiniani 2, 35100 Padova, Italy; E-Mail: vvindigni@unipd.it; 3 Department of Periodontology, School of Dentistry, University of Padova; Via Venezia 90, 35100 Padova, Italy; E-Mail: eriberto.bresssan@unipd.it; 4 Unit of Neurosurgery; University of Padova, Via Giustiniani 2, 35100 Padova, Italy; E-Mails: alessandropuppa@unipd.it (A.D.P.); Domenico.valella@unipd.it (D.D.); 5 Department of Prosthodontics, Dental Clinic, School of Dentistry, University of Milan, 21120 Milan, Italy; E-Mail: lops2@libero.it; 6 Department of Experimental and Diagnostic Medicine, Section of General Pathology, Interdisciplinary Center for the Study of Inflammation (ICSI) and LTTA center, University of Ferrara, 44100 Ferrara; Italy; E-Mail: pnp@unife.it

**Keywords:** adipose derived stem cells, skin, adipose tissue, stem cells, Schwann cell, karyotypes

## Abstract

Recently, we have described a simple protocol to obtain an enriched culture of adult stem cells organized in neurospheres from two post-natal tissues: skin and adipose tissue. Due to their possible application in neuronal tissue regeneration, here we tested two kinds of scaffold well known in tissue engineering application: hyaluronan based membranes and fibrin-glue meshes. Neurospheres from skin and adipose tissue were seeded onto two scaffold types: hyaluronan based membrane and fibrin-glue meshes. Neurospheres were then induced to acquire a glial and neuronal-like phenotype. Gene expression, morphological feature and chromosomal imbalance (kariotype) were analyzed and compared. Adipose and skin derived neurospheres are able to grow well and to differentiate into glial/neuron cells without any chromosomal imbalance in both scaffolds. Adult cells are able to express typical cell surface markers such as S100; GFAP; nestin; βIII tubulin; CNPase. In summary, we have demonstrated that neurospheres isolated from skin and adipose tissues are able to differentiate in glial/neuron-like cells, without any chromosomal imbalance in two scaffold types, useful for tissue engineering application: hyaluronan based membrane and fibrin-glue meshes.

## 1. Introduction

Interest about novel stem cell-based therapies has exponentially been increasing over the past years, not only in the scientific community but also within the society. Indeed, stem cells seem to give the best chance for human tissue engineering, and particularly, mesenchymal stem cells (MSCs) represent a great tool in regenerative medicine because of their ability to differentiate into a variety of specialized cells in addition to their immuno-privileged characteristics [1,2].

Mesenchymal stem cells are adherent stromal cells able to self-renew and differentiate into a wide variety of cells and tissues. Mesenchymal stem cells can be isolated from different sources: amnion, placenta, bone marrow, umbilical cord and cord blood, adipose tissue and dental pulp are the most common ones; moreover, these cells are available in virtually all post-natal tissues. As adult stem cells, mesenchymal stem cells have not the tumorigenic potential as their embryonic correlatives and possess other unique characteristics such as their almost null immunogenicity. Moreover, these cells seem to be immunosuppressive *in vitro* [3,4].

Mesenchymal stem cells can differentiate into non-mesenchymal lineages as a result of their great plasticity.

While MSCs were initially defined by their ability to differentiate into cells of mesodermal origin, recent studies have provided support for their capacity to differentiate into cells from all three germ layers [5]. In addition, MSCs have other characteristics making them attractive modalities for treating human disease. These cells are described as MHC II negative cells, lacking co-stimulatory molecules such as CD40, CD80 and CD86, which permit allogenic transplantation without immunosuppression [6]. Furthermore, they can be easily isolated from an autologous source, enabling easy accessibility for therapeutic intent. MSCs can provide therapeutic benefit through the secretion of specific cytokines, *ex vivo* genetic modification and direct cell-cell contact. As such, these cells have been studied for their use in diverse diseases ranging from genetic disorders to tissue ischemia [7].

In this context, the ability to generate Schwann cells and their precursors from adult non-neural tissues such as bone marrow, adipose tissue and skin is of significant clinical interest [8]. Schwann cells can provide beneficial therapeutic effects in both the peripheral and central nervous systems by remyelination, provision of trophic support, and a role in promoting axon regeneration [9]. A number of recent reports have suggested that cells with a glial phenotype can be generated from stem cells derived from both bone marrow and skin [10–12]. However, the identity of the original cells that give rise to putative Schwann cells remains poorly defined. In both cases, it has been proposed that Schwann cells are generated from precursors within these tissues. In the case of bone marrow and adipose tissue, this differentiation started from MSC; in the case of skin, Schwann cell differentiation has been reported from skin-derived precursor cells (SKPs). SKPs are defined as multipotent precursor cells that can grow as self-renewing precursors under substrate-free conditions in media specialized for neural precursor propagation [13,14]. SKPs have also been shown to possess mesenchymal as well as neuroglial potential [15].

In light of such considerations, one possible clinical application of this ability is undoubtedly the *in vitro* or *in vivo* studies of the mechanism for the treatment of neurodegenerative states, such as Parkinson’s disease, Hungtinton’s disease, multiple sclerosis and Alzheimer’s disease [16–18].

In order to reach these goals, other necessary strategies, such as tissue engineering and regenerative medicine, should be combined with stem cell biology. Indeed the ability of the scaffolds to support 3D growth, differentiation or cell-release *in situ* are well known [19,20].

Tissue Engineering and regenerative medicine aim to investigate the deposition, growth and remodeling of tissues by drawing together approaches from a range of disciplines [21].

Recent developments in the multidisciplinary field of tissue engineering have yielded many novel tissue replacements and implementation strategies. Scientific advances in biomaterials, stem cell isolation, growth and differentiation factors and biomimetic environments have created unique opportunities to fabricate tissues in the laboratory. One of the major challenges now facing tissue engineering is to optimize 3D functional recovery, a factor dependent on 3D structural complexity, as well as the biomechanical and functional stability of the laboratory-grown tissues destined for transplantation [22].

A variety of synthetic and naturally derived materials may be used for tissue engineered scaffolds. Synthetic materials include poly(ethylene oxide), poly(vinyl alcohol), whereas representative naturally derived polymers include agarose, alginate, chitosan, collagen, fibrin, gelatin, and hyaluronic acid [23].

Selection or synthesis of the appropriate hydrogel scaffold materials is governed by the physical property, the mass transport property, and the biological interaction requirements of each specific application. These properties or design variables are specified by the intended scaffold application and environment into which the scaffold will be placed [24–27].

In light of these considerations, our lab recently merged its great experience in biomaterials with its expertise in tissue engineering to obtain the *in vitro* commitment of adult mesenchymal stem cells—skin and adipose tissue derived—into a neuronal phenotype [28,29].

With our previous results [14,15], we established a protocol for the isolation and amplification *in vitro* of a population of adult neuronal stem cells from two tissues: skin and adipose tissue.

We can summarize that the principal culture requirements for the successful formation of neurospheres *in vitro* from either skin or adipose tissue are the following: (1) cells isolated from the tissue must be cultured without coating chamber slides; (2) the proliferative medium must contain serum; and (3) both EGF and FGF2 must be added to the media.

With this protocol, skin derived stem cells, termed skin-derived precursors (SKPs), were isolated and expanded from human skin-adipose tissue and differentiated into neural and mesodermal progeny, including cell types that are never found in skin or in adipose tissue, such as neurons.

With a very simple protocol, putative adult human neural crest precursors, firstly in form of proliferative neurospheres, then involving only the use of two similar mitogens (FGF2 and EGF) in two weeks we were able to isolate and expand populations of adult human CNS precursors and in cells with a neural-glial like morphology.

Surprisingly, the amount and the neurological properties of these stem cells are comparable. The encouraging results obtained in monolayer cultures, drove us to investigate cell commitment in a 3D system. In order to detect the best material, we compared two scaffolds: hyaluronan based and fibrin based scaffolds. Genetical, biochemical, molecular biology and morphological approaches have been used to better characterize the influence of the nature of the biomaterial on stem cells.

## 2. Results and Discussion

### 2.1. Biomaterials

#### 2.1.1. Hyaluronan

Biomaterials used in the present study were derived from the total esterification of hyaluronan (synthesized from 80–200 kDa sodium hyaluronate) with benzyl alcohol, referred to as HYAFF-11™. The final product is an uncross-linked linear polymer with an undetermined molecular weight; it is insoluble in aqueous solution yet spontaneously hydrolyzes over time, releasing benzyl alcohol and hyaluronan. HYAFF-11™ was used to create non-woven meshes (NW) (50 μm-thick fibers; specific weight of 100 g/m^2^). These devices were obtained from Fidia Advanced Biopolymers (FAB, Abano Terme, Italy).

#### 2.1.2. Fibrin Glue

Fibrin glue is a synthetic substance made to create *in vitro* a fibrin clot. It is based by fibrinogen and thrombin that are injected through one head into the site of a fibrin tear. Thrombin acts as an enzyme and converts the fibrogen into fibrin between 10 and 60 s. These devices were obtained from Baxter (Baxter AG, Vienna, Austria).

### 2.2. Cell Cultures

Skin biopsies and adipose tissue samples were obtained during abdominoplastic procedures from the abdominal region of a same set of seven patients (aged 35–58) who had given written consent.

Cell extraction was performed according to previously published protocols for skin [11] and adipose tissue [12]. Briefly, the full-thickness skin biopsies (3 cm × 1 cm) were accurately cleared of the subcutaneous tissue and cut into 1 mm × 1 mm pieces. These were suspended in DMEM (Biochrom AG) and Whartington’s collagenase (Sigma) 80 U for overnight digestion. Adipose tissue was collected and cut into small pieces, suspended in DMEM with collagenase type 2 (Sigma) for 90 min in slow agitation. After centrifugation for 5 min at 1000 rpm the supernatant was removed, the pellet was resuspended with a Pasteur pipette and the suspension was passed through a 70-μm cell strainer (BD Biosciences, Mississauga, Ontario, Canada). The strained cell suspension was centrifuged, resuspended in proliferating medium (DMEM-HAM’s F12 (3:1), penicillin/streptomycin 1%, EGF (20 ng/μL), FGF (40 ng/μL), FBS 10%) after removal of the supernatant and transferred to a 25-cm^2^ tissue culture flask (BD Biosciences). Cells were cultured at 37°C with 5% CO_2_ atmosphere.

Proliferative Medium: DMEM-HAM’s F12, 3:1, penicillin/streptomycin 1%, EGF (1000×), FGF (100×), FBS (10%).

Neuronal Differentiative Medium: DMEM-F12, 3:1, 1% FBS, 2% b27 serum free supplement, 10 μg/mL NGF, antibiotics [30].

Glial Differentiative Medium: DMEM-F12, 3:1, containing 1% FBS plus 1% N2 supplement, 4 μM forskolin, and 10 ng/mL heregulin β [31].

Non Differentiative Medium: Non hematopoietic (NH) stem cell expansion media (Miltenyi Biotec, Bergish Gladbach, Germany).

### 2.3. Morphological Analysis

Cultures were layered over glass slides, fixed with absolute acetone for 10 min at room temperature and cryopreserved at −20 °C until use. S100; GFAP; nestin; bIII tubulin; CNPase were visualized with immunofluorescence. Briefly, after non-specific antigen sites were saturated with 1/20 rabbit serum in 0.05 M maleate TRIZMA (Sigma; pH 7.6) for 20 s, 1/100 mouse anti-human S100 (Sigma) was added to the samples. After incubation, immunofluorescence staining was performed by means of fluorescein secondary antibody [32–35]. As negative control, reactions without primary antibody were used. No background in any sample has been revealed. Stem cells cultured in non differentiative medium have been used as negative control too.

### 2.4. Morphological Analysis

Cultures were layered over glass slides, fixed with absolute acetone for 10 min at room temperature and cryopreserved at −20 °C until use. S100; GFAP; nestin; bIII tubulin; CNPase were visualized with immunofluorescence. Briefly, after non-specific antigen sites were saturated with 1/20 rabbit serum in 0.05 M maleate TRIZMA (Sigma; pH 7.6) for 20 s, 1/100 mouse anti-human S100 (Sigma) was added to the samples. After incubation, immunofluorescence staining was performed by means of fluorescein secondary antibody [17–19].

### 2.5. SEM

Scaffolds were fixed, postfixed, dehydrated, and sputter coated with gold as standard protocols. Samples were examined using a Philips XL-40 scanning electron microscope (FEI, Eindhoven, The Netherlands).

### 2.6. Cytogenetic Analysis

At p3 cells were exposed to colchicines (Sigma Chemicals, St. Louis, MO, USA) for 6 h, detached with trypsin (Lonza, Milano, Italy), washed with phosphate-buffered saline (Lonza, Milano, Italy), subjected to sodium citrate 1% for 15 min at 37 °C, and fixed and spread according to standard procedures. Metaphases of cells were Q-banded and karyotyped in accordance with the international System for Human Cytogenetic Nomenclature recommendations. Twenty-five metaphases were analyzed for three expansions.

### 2.7. MTT Test

MTT-based (thiazolyl blue) cytotoxicity test was performed as described in Denizot and Lang [35], with minor modifications. The test is based on mitochondria viability, *i.e*., only functional mitochondria can oxidize an MTT solution, giving a typical blue-violet end product. This assay is an indirect method to assess cell growth and proliferation since the optical density (OD) value can be correlated to the cell number. All samples were examined after 3, 7 and 14 days of culture.

### 2.8. Statistical Analysis

The one-way analysis of variance (Anova test) was used for data analyses. Repeat measurement analysis of variance (Re-ANOVA) and paired t tests were used to determine if there were significant changes (*p* < 0.05). * *p* < 0.05; ** *p* < 0.01; *** *p* < 0.001: Repeatability was calculated as the standard deviation of the difference between measurements.

## 3. Experimental Section

### 3.1. Chromosome Stability

Chromosome stability of cell cultures has been detected by means of the karyotipe analyses. The karyotype describes the number of chromosomes, and what they look like under a light microscope. The chromosomes are depicted (by rearranging a microphotograph) in a standard format known as a *karyogram*: in pairs, ordered by size and position of centromere for chromosomes of the same size.

The normal human karyotype contains 22 pairs of autosomal chromosomes and one pair of sex chromosomes. Normal karyotypes for females contain two X chromosomes. In our experiments all the adipose derived stem cells are 46-XX since they derived from female abdominoplastic adipose tissue part, males have both an X and a Y chromosome and are denoted 46-XY. Whereas skin derived stem cells used in our experiments have an XY set of sexual chromosomes since they derived from male abdominoplastic dermal tissue parts. Any variation from the standard karyotype may lead to developmental abnormalities.

**Figure 1:**
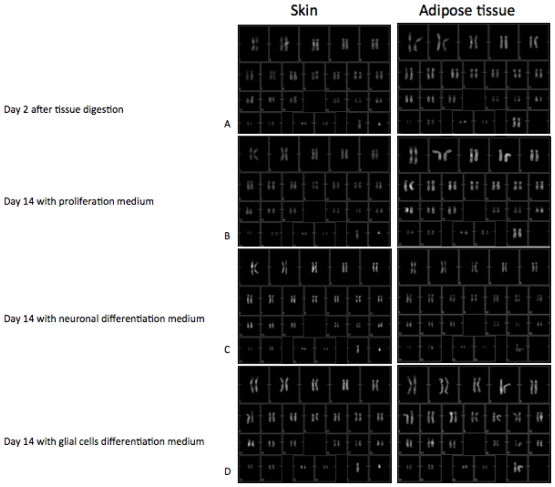
Karyotypes of skin-derived precursors (SKPs) and adipose derived stem cells (ADSc) have been performed during the principal phase of the protocol: 2 days after the digestion of the tissue; 14 days after a treatment in proliferative medium; 14 days after a treatment in differentiative medium (Figure 1c for the neuronal and Figure 1d for the Glial one).

Karyotypes of skin-derived precursors and adipose derived stem cells have been performed during the main phases of the protocol:

Two days after the digestion of the tissues (Figure 1a), with the aim to establish the presence of any chromosome imbalance in the original status of the cells.

Fourteen days after a treatment in proliferative medium (Figure 1b), with the aim to verify if the factors present in the proliferative medium were able to induce some alterations in the normal sets of chromosomes.

Fourteen days after a treatment in differentiative medium (Figure 1c for the neuronal medium and Figure 1d for the Glial one), in order to verify if the new factors associated to the long term culture (28 days after the digestion) were able to induce some chromosomal alterations.

As reported in all the figures, cells cultured in all the conditions tested give rise to normal karyotypes. No chromosomal imbalances such as trysomies or monosomies in chromosome number are evident, nor are translocation/deletion/duplication on chromosome structures present.

### 3.2. Morphological Features in Monolayer Conditions

After 14 days of culture in proliferative medium, skin-derived precursors and adipose derived stem cells have been treated with the specific differentiative medium.

Markers for both neuronal and Schwann cells have been detected by immuno-staining in all cell types with the aim to test the correct commitment.

**Figure 2:**
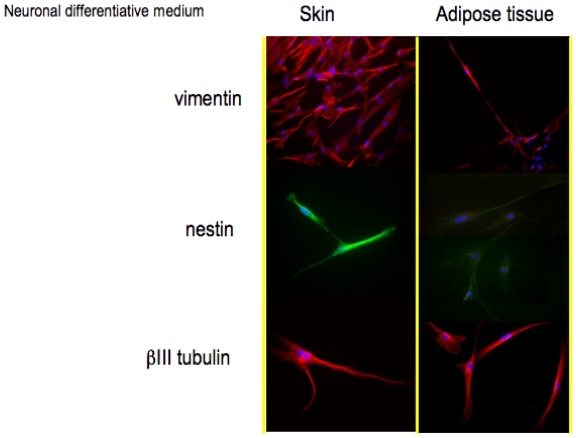
Immunofluorescence stainings to detect Vimentin, nestin, βIII tubulin on skin and adipose tissue-derived stem cells, in presence of neuronal differentiative medium.

In Figure 2, histological detection for the markers associated to a neuronal phenotype is reported.

Vimentin (57 kDa) is the most ubiquitous intermediate filament protein and the first to be expressed during cell differentiation. Vimentin is present in a wide variety of mesenchymal cell types and in many cells from the neural crest. The positive staining for vimentin in both skin-derived precursors and adipose derived stem confirms their staminal state.

The second protein whose expression is related to a neuronal phenotype is nestin. Nestin, a type VI IF protein, is a protein marker for neural stem cells since it is mostly expressed in nerve cells where it is implicated in the radial growth of the axon. Nestin is expressed in dividing cells during the early stages of development in the central nervous system. Upon differentiation, nestin becomes down-regulated and is replaced by tissue-specific IF proteins; in our monolayer cell cultures, both skin-derived precursors and adipose derived stem cells (Figure 2, second line) in presence of neuronal medium express nestin. During gliogenesis, nestin is replaced by IF, such as glial fibrillary acidic protein (GFAP). In our cultures, indeed, no cells express GFAP if cultured with neuronal medium (data not shown).

The last protein tested for the validation of a neuronal phenotype is βIII tubulin, a protein abundant in the central and peripheral nervous systems (CNS and PNS) where it is prominently expressed during fetal and post-natal development. Also in this case, all the cells show a good expression of this neuronal protein.

**Figure 3:**
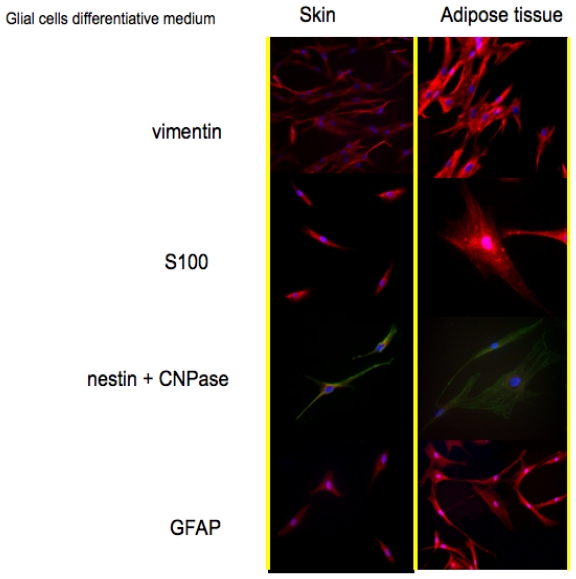
Immunofluorescence stainings to detect Vimentin, S100; CNP; nestin; GFAPase on skin and adipose tissue-derived stem cells in presence of glial differentiative medium.

Protein expression pattern during cultures in Glial cells medium is reported in Figure 3. Also for this treatment vimentin has been selected as a marker for stem cells. As reported in the first line, also in this case both cell types are positive. The co-expression of vimentin with GFAP; is typical of glial cells; an event that is well depicted in the fourth line of Figure 3.

Vimentin is frequently included in the so-called primary panel (together with CD45, cytokeratin, and S-100 protein). In our panel we included the detection of S100, a calcium binding protein normally present in cells derived from the neural crest (Schwann cells and glial cells), in mesenchymal derived cells such as chondrocytes, adipocytes, and in dendritic cells. In line 2 of Figure 3, it is evident that both skin-derived precursors and adipose derived stem cells express S100 as confirmed by the red fluorescent staining.

Nestin and CNPase:CNPase (2′,3′-Cyclic Nucleotide 3′-Phosphodiesterase) is expressed at high levels by oligodendrocytes in the CNS and by Schwann cells in the peripheral nervous system (PNS), and it is virtually absent in other cell types. Its co-expression with nestin confirms the commitment of skin-derived precursors and adipose derived stem cells into a correct phenotype.

### 3.3. Proliferation on 3D Cultures

**Figure 4:**
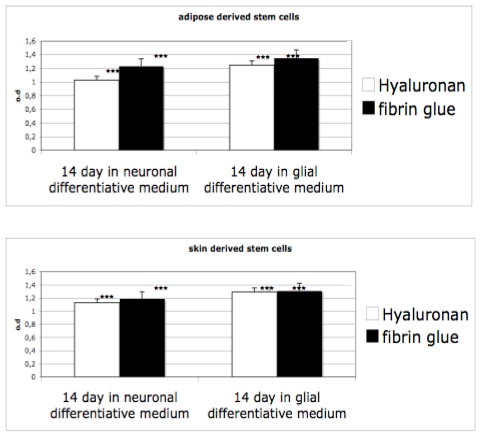
Proliferation test (by means of MTT) of adipose derived stem cells and skin derived stem cells into hyaluronan (white bars) and fibrin glue (black bars) after 14 days in differentiative medium. The one-way analysis of variance (Anova test) was used for data analyses. Repeat measurement analysis of variance (Re-ANOVA) and paired t tests were used to determine if there were significant changes (*p* < 0.05). * *p* < 0,05; ** *p* < 0.01; *** *p* < 0.001: Repeatability was calculated as the standard deviation of the difference between measurements.

After induction of neurosphere formations in monolayer conditions, cells have been seeded into hyaluronan and fibrin based scaffolds. Proliferation ability of cells differentiated has been evaluated by means of an MTT test. As reported in Figure 4, the proliferation ability of the cells in both materials, grown in presence of proliferative medium, is evident. When cells are cultivated in differentiative medium (for glial or for neuro one) the rate decreases. Indeed, in this case, a selection of cells occurs (only neursphere are able to survive) and the cells acquire a well defined phenotype. Interestingly, no significant difference is evident between the scaffolds.

### 3.4. Gene Expression on 3D Cultures

**Figure 5:**
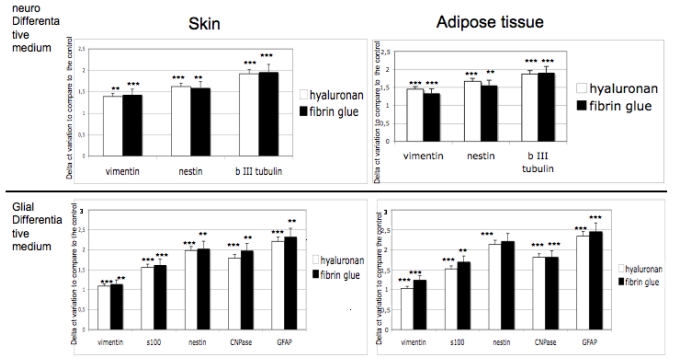
Gene expression analyses of adipose and skin derived stem cells after 14 days of culture in differentiative medium into hyaluronan (white bars) and into fibrin glue (black bars) based scaffolds. The one-way analysis of variance (Anova test) was used for data analyses. Repeat measurement analysis of variance (Re-ANOVA) and paired t tests were used to determine if there were significant changes (*p* < 0.05). * *p* < 0.05; ** *p* < 0.01; *** *p* < 0.001: Repeatability was calculated as the standard deviation of the difference between measurements.

Commitment of adult stem cells into 3D scaffolds has been tested by means of gene expression analyses. Expression of markers for neural phenotypes such as vimentin, nestin, βIII tubulin and for glial commitment such as vimentin, S100, nestin, CNPase, GFAP, has been detected after 14 days of 3D cultures in both scaffolds. As controls, adipose derived stem cells and skin derived stem cells cultured with no differentiative medium have been used. In Figure 5, expression of markers in presence of differentiative medium, compared with the expression in no differetiative medium, are reported.

Results obtained confirm that in both scaffolds the correct commitment occurred. Expression of specific markers is indeed always detectable in hyaluronan or in fibrin glue based scaffolds and no significant difference between the biomaterials is evident.

### 3.5. Electron Microscopy Analyses

Skin-derived precursors and adipose derived stem cells have been cultured in scaffolds based on fibrin glue and hyaluronic acid.

The thickness and the material of the materials did not allow the immunohistochemical staining previously obtained. For this reason we performed the morphological evaluation with SEM.

**Figure 6:**
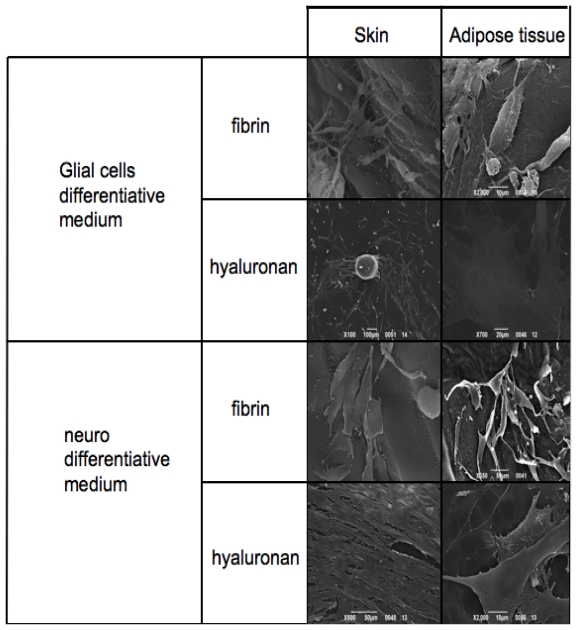
SEM analyses of skin and adipose tissue-derived stem cells in presence of glial/neuronal differentiative medium in hyaluronan/fibrin glue based scaffolds.

Figure 6 reports SEM analyses of both skin-derived precursors and adipose derived stem cells treated with neuronal and Schwann cell medium on fibrin glue and hyaluronic acid based scaffolds.

The first panel is related to the phenotype acquired by the cells in presence of Glial cell differentiative medium. Skin-derived precursors and adipose derived stem cells show a classical shape with a triangular central group and typical somata and two or more pronounced dendrite or axon-like processes on fibrin glue. This phenotype is present also when cells are cultured in hyaluronan based scaffold. In this case adipose derived stem cells assume a flat and large conformation.

When cells are cultured in presence of neuronal medium it is possible to conclude with the same observations: both skin-derived precursors and adipose derived stem cells are able to grow in both scaffolds acquiring the correct phenotype. Notably in hyaluronan scaffolds, cells may acquire a more fibroblastic-like feature reaching confluence in a few days.

## 4. Discussion

At present, great progress and breakthroughs have been achieved in stem cell-based therapies for neurodegenerative disorders. However, the development of stem cell-based therapies is still at an early stage. Many basic issues remain to be resolved. Although embryonic stem cells seem to have an unrestricted potential to differentiate towards neuroectodermal phenotypes, the differentiation *in vitro* is extremely random. Embryonic stem cells can give rise to teratomas and immune rejection response after transplantation. The risk of teratoma from embryonic stem cells, as well as the consequences of introducing new genes in stem cell-derived neurons, should be carefully evaluated [20]. An alternative source is neural stem cells for which the isolation process is standardized. Neuronal stem cells can differentiate into all of the major neural cell types *in vivo* and *in vitro*. When transplanted into the brain they are able to survive, migrate and integrate in a functionally active way. Stem cells isolated from adult brain as neurospheres generate fewer neurons than those isolated from embryonic or fetal brain, both in transplantation cases and in differentiating conditions *in vitro* [36,37]. Recently, great scientific success has been obtained from mesenchymal stem cells, isolated from different adult tissues.

While embryonic stem cells and neuronal stem cells have great potential, mesenchymal stem cells also provide hopeful possibilities for clinical application, since they can be efficiently expanded *in vitro* and it is possible to acquire a therapeutic scale of induced cells. In addition, transplantation of mesenchymal stem cells-derived cells should pose fewer ethical problems by preventing stem cell controversy, since bone marrow transplantation has already been widely performed. Thus, from the point of view of the cell-based therapy, it is desirable to develop a systematic induction system to obtain large amounts of useful cells. Indeed, practical application to human degenerative diseases depends on the ability to control their differentiation into functional cells with high efficiency and purity.

Recently we have demonstrated [14,15] that we are able to induce the *in vitro* commitment into a glial and neuronal like phenotype of adult stem cells derived from adipose tissue and skin.

Due to these encouraging results, in the present work we translated our experience from a monolayer condition into 3D conditions, a more physiological environment. Firstly we checked the safety of our differentiation methods performing a detailed genomic analysis (by means of caryotiping) of cultures throughout all the *in vitro* steps (Figure 1). The absence of any structural alteration confirmed the safety to differentiate mesenchymal stem cells.

The second step has been represented by the immunostaining of specific markers for neuronal and glial commitment (Figures 2 and 3). The expression and the morphology of the cells confirmed the correct phenotype was used.

At this stage, we seeded the neuspheres into the two selected scaffolds: hyaluronan and fibrin glued based meshes. We then performed the proliferation test (Figure 4) to test the biocompatibility of the scaffolds and gene expression to test the commitment (Figure 5) of the cells. As it has been well evidenced, the scaffolds also support the growth and the differentiation process. The gene expression is clear, as reported in Figure 5, as all the specific markers have been detected. Finally, morphological features, such as the distribution into the scaffolds, have been analyzed with electron microscopy. Figure 6 reports on cell distribution and the morphological features of the cells in the two scaffolds.

## 5. Conclusion

In the present work, we showed the high ratio and specific induction of glial cells and neurons from adult stem cells derived from skin and adipose tissues. Moreover, we thoroughly checked the safety of our protocols.

In conclusion, with classical tissue engineering techniques, we tested two scaffolds based on hyaluronan and fibrin glue in order to confirm if the neuronal and glial commitment of the mesenchymal stem cells derived from adipoe tissue and skin could be feasible.

The encouraging results clearly showing morphological features related to a neuronal and glial-like phenotype, impel us to transfer our 3D cultures to an *in vivo* model.
